# Comprehensive Analysis of the Correlation Between Pyroptosis-Related LncRNAs and Tumor Microenvironment, Prognosis, and Immune Infiltration in Hepatocellular Carcinoma

**DOI:** 10.3389/fgene.2022.867627

**Published:** 2022-04-26

**Authors:** Guangzhen Qu, Dong Wang, Weiyu Xu, Wei Guo

**Affiliations:** Department of General Surgery, Beijing Friendship Hospital, Capital Medical University, National Clinical Research Center for Digestive Diseases, Beijing, China

**Keywords:** hepatocellular carcinoma, pyroptosis, lncRNAs, risk signature, tumor environment, immunotherapy

## Abstract

**Background:** Accumulating evidence shows that pyroptosis plays a crucial role in hepatocellular carcinoma (HCC). However, the relationship between pyroptosis-related long non-coding RNAs (lncRNAs) and HCC tumor characteristics remains enigmatic. We aimed to explore the predictive effect of pyroptosis-related lncRNAs (PRLs) in the prognosis of HCC.

**Methods:** We comprehensively analyzed the role of the PRLs in the tumor microenvironment and HCC prognosis by integrating genomic data from patients of HCC. Consensus clustering analysis of PRLs was applied to identify HCC subtypes. A prognostic model was then established with a training cohort from The Cancer Genome Atlas (TCGA) using univariate and least absolute shrinkage and selection operator (LASSO) Cox regression analysis. Further, we evaluated the accuracy of this predictive model using a validation set. We predicted IC50s of commonly used chemotherapeutic and targeted drugs through the R package pRRophetic.

**Results:** Based on pyroptosis-related lncRNAs, a prognostic risk signature composed of seven PRLs (MKLN1AS, AL031985.3, SNHG4, GHRLOS, AC005479.2, AC099850.4, and AC026412.3) was established. For long-term prognosis of HCC patients, our model shows excellent accuracy to forecast overall survival of HCC individuals both in training set and testing set. We found a significant correlation between clinical features and the risk score. Patients in the high-risk group had tumor characteristics associated with progression such as aggressive pathological grade and stage. Besides that, gene set enrichment analysis (GSEA) showed that cell cycle and focal adhesion were significantly enriched in the high-risk group.

**Conclusion:** The association of the risk model constituted by these seven pyroptosis-related lncRNAs with clinical prognosis, tumor microenvironment, chemotherapy and small molecule drugs was evaluated. Our study provides strong evidence for individualized prediction of prognosis, shedding light on immunotherapy in HCC patients.

## Introduction

The incidence of hepatocellular carcinoma (HCC) continues to increase worldwide, with an estimated one million cases per year by 2025, posing a challenge to global health (Llovet et al., 2021). Heterogeneity exists in tumor cells within and among tumors and the varied landscapes of the tumor microenvironment (TME), Which is directly associated with poor prognosis of HCC patients (Cao et al., 2020). Therapies such as surgery, chemoradiotherapy, liver transplantation, and radiofrequency ablation are limited; hence, the overall prognosis prospects of HCC remain unsatisfactory (Yu et al., 2021a). Pyroptosis is a newly discovered programmed cell-death pathway that occurs via classical and non-classical pathways, with continuous cell expansion until the cell membrane ruptures and the releases of cellular contents followed by activation of a strong inflammatory response (Rogers et al., 2017; Broz et al., 2020).

Pyroptosis is involved in the progression of multiple types of tumors, including HCCs. Hu et al. Hu et al. (2021) showed that six gasdermin (GSDM) family members involved in pyroptosis play an indispensable role in HCC. Higher GSDME expression is positively correlated with shorter overall survival (OS) and disease-specific survival (DSS) in HCC patients. Shen et al. (Shen et al., 2021) demonstrated that metformin attenuates HCC cell growth by inducing pyroptosis through relying on FOXO3 to activate NLRP3 transcription. The functions and associations of additional pyroptosis genes in HCC development, immune infiltration, and prognosis remain to be determined. Long non-coding RNAs (lncRNAs), a class of RNAs with more than 200 nucleotides in length and do not encode proteins (Mercer et al., 2009). LncRNAs are key gene expression regulators involved in the progression of several diseases, including cancer, and are associated with tumor proliferation, differentiation, apoptosis, and metastasis (Yu et al., 2021b). Studies have shown that lncRNAs are involved in HCC progression; for instance, lncRNA Ptndt promotes HCC cell proliferation by binding to HuR protein (Huang et al., 2019). LncRNA ATB upregulates ZEB1 and ZEB2 by binding to miR-200, inducing epithelial-mesenchymal transition and HCC invasion (Yuan et al., 2014). The role of pyroptosis-modified lncRNAs has been demonstrated in lung, breast, and head and neck cancers (Liu et al., 2019; Wu et al., 2021; Zhu et al., 2021). However, the correlation between pyroptosis-modified lncRNAs and the TME, immune infiltration, and immune checkpoint genes in HCC remains unclear. Therefore, it is vital to determine the role of pyroptosis-modified lncRNAs in HCC, which will provide novel insights into HCC treatment.

TME is the site and material basis for tumorigenesis, progression, and metastasis, and is a dynamic system composed of immune cells, tumor-associated fibroblasts, endothelial cells, and other non-bioactive environmental factors (Hinshaw and Shevde, 2019). HCC cells can remodel the tumor immunosuppressive microenvironment by secreting cytokines and chemokines (Han et al., 2019). Immune checkpoint inhibitors (anti-PD1, anti-PD-L1, and anti-CTLA4) have shown efficacy in the treatment of patients with advanced tumors (Motzer et al., 2015). However, the immunosuppressive TME of HCC only allow immunotherapy to benefit a limited proportion of patients with HCC. Several immune-related lncRNAs play crucial roles in tumor immunotherapy resistance (Zhou et al., 2019). Investigating the association of pyroptosis-related lncRNAs (PRLs) with the HCC TME, immune gene expression, and immune infiltration may provide a different perspective on HCC immunotherapy.

**FIGURE 1:**
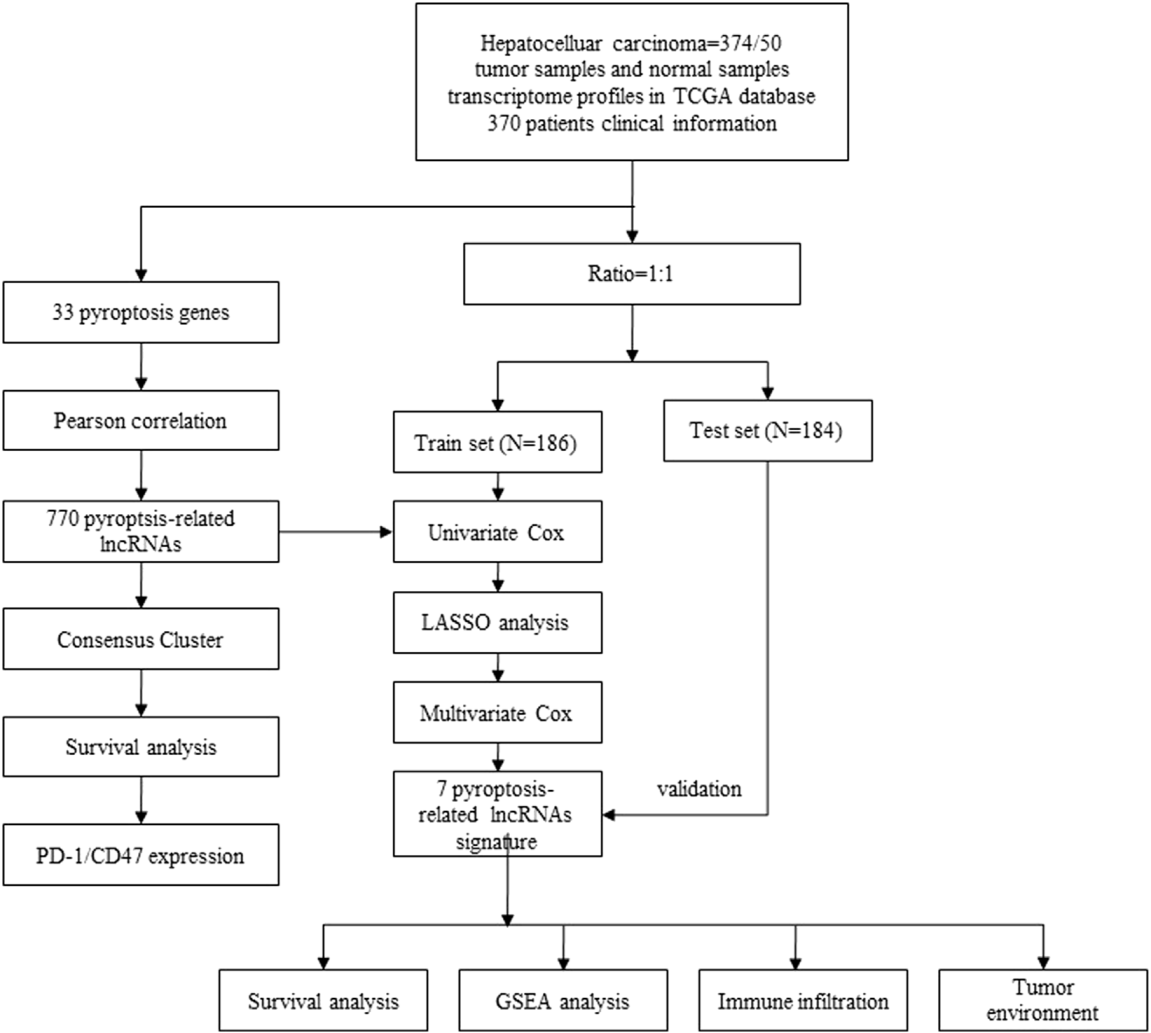
Flow chart of our study based on The Cancer Genome Atlas (TCGA) datasets.

In this study, we explore the association between PRLs and the TME, immune infiltration, and prognosis of HCC. A signature based on PRLs was obtained to perform prognostic risk stratification in patients with HCC. We also evaluate the expression of PRLs and their role in HCC immunotherapy. Our results reveal the potential connection between pyroptosis, prognosis, the immune microenvironment, and the response to immunotherapy in HCC patients. A flowchart of our study is presented in Figure 1.

## Materials and Methods

### Data Acquisition

The RNA sequence profiles of 425 HCC samples (375 tumor tissue and 50 normal samples) and the corresponding survival information were downloaded from The Cancer Genome Atlas (TCGA) database. The gene transcriptome information was normalized by fragment per kilobase of exon model per million (FPKM). The “combat” algorithm from the R package “sva” was used to remove batch effects. The copy number variants (CNV) profiles of HCC samples were downloaded from UCSC Xena (https://xena.ucsc.edu/). After excluding samples from HCC patients with missing clinical information, 370 HCC samples were included into our study. The “caret” package was utilized to randomly split HCC samples into train and test cohorts in a 1:1 ratio.

### Consensus Clustering Analysis for PRLs

According to previous studies, 33 pyroptosis-related genes (PRGs) were identified (Supplementary Table S1) (Man and Kanneganti, 2015; Karki and Kanneganti, 2019; Ye et al., 2021). LncRNA annotations and protein-coding genes were obtained from the Ensembl human genome browser GRCh38 (Cunningham et al., 2019). According to Pearson correlation analysis, the pyroptosis-related lncRNAs were filtered using the following criteria: “correlation coefficient >0.4” and “*p* < 0.001” (Hinshaw and Shevde, 2019). Then univariate Cox regression analysis was performed to screen PRLs in the training set that potentially correlated with HCC prognosis. Based on the result of univariate Cox regression analysis, the “ConsensusClusterPlus” ^3^ was used to categorize the HCC cohorts into distinct pyroptosis modification patterns (subtypes), and 1,000 repeats were performed to ensure the stability of classification. Consensus clustering is a popular algorithm, which was extensively utilized in medical studies (Liu et al., 2021a; Liu et al., 2021b; Liu et al., 2022a; Liu et al., 2022b).

### Clinical Correlations Between PRLs and Different Subtypes

We compared clinically relevant information, such as gender, stage, T, N, M stage, and grade, between different subtypes of HCC patients. Here, we aimed to determine the correlation between clinical information and subtypes according to potential PRLs. Further, the overall survival (OS) of different subtypes was compared.

### Pyroptosis-Related LncRNAs Signature Was Constructed and Validated

Through univariate Cox regression analysis and least absolute shrinkage and selection operator (LASSO) regression analysis results, we obtained the prognostic risk signatures composed of seven PRLs in the training set. The risk scores of HCC patients in both the training and testing sets were calculated using the following formula:

$$\text{Risk score }=\sum_{i=1}^{n}codfi*xi,$$

where codfi represents the coefficient and xi the relative expression value of each PRLs.

The HCC patients in the training set (*n* = 185) were categorized into either low-risk (PRL score < median value) or high-risk (PRL score > median value) groups based on the median risk score; parallelly, patients in the validation set were also into either low-risk and high-risk groups. Univariate and multivariate Cox regression analyses were performed to evaluate the independent prognostic significant of the pyroptosis-related lncRNAs prognostic signature. Kaplan–Meier curve was performed to determine the impact of prognostic PRLs signature on OS. The 1-, 3-, and 5-years receiver operating characteristic (ROC) curves were constructed to evaluate the accuracy of prognostic PRLs signature.

### Immune Checkpoint-Related Gene Expression and Immune Infiltration Analysis

CD47 is a transmembrane protein upregulated in various tumors. Several clinical trials on CD47-targeting agents are ongoing that have shown promising results (Chester et al., 2015; Liu et al., 2015). Kim et al. demonstrated that CD47 is upregulated in HCC tumor tissues and correlated with shorter recurrence-free survival (Kim et al., 2021). The differences in PD-1 and CD47 expression between the clusters and normal and tumor samples were also analyzed. The association between PD-1 expression and PRLs was depicted using the “corrplot” package. CIBERSORT was utilized to analyze the abundance of 22 immune cell types identified in each tumor sample from the TCGA database. ESTIMATE is an algorithm used to evaluate tumor purity and immune/stromal infiltration score in tumor samples. We used the “estimate” package to assess the immune, stromal, and ESTIMATE scores between two HCC patterns.

### Immunity Analysis for Low-Risk and High-Risk Groups

The abundance of immune cell infiltration in low-risk and high-risk groups was calculated using the CIBERSORT(It deconvolutes the expression matrix of human immune cell subtypes based on the principle of linear support vector regression), CIBERSORT-ABS (Quantification of immune cell components using deconvolution of gene expression information and sophisticated algorithms), QUANTISEQ (Which based on deconvolution algorithms that make predictions about the composition of different classes of immune cells in tumor samples), XCELL (It is a webtool that performs cell type enrichment analysis based on gene expression data of 64 immune and stromal cell types and enables researchers to reliably delineate the cellular heterogeneity landscape), MCPcounter (It analyzes the abundance of tumor immune and stromal cell infiltration based on gene expression), EPIC (Estimation of the proportion of immune and tumor cells from gene expression data using deconvolution) and TIMER (Which uses RNA sequencing data to calculate the extent of immune cell infiltration in tumor tissue) algorithms.

### Function and Immunotherapy Analysis in Low-Risk and High-Risk Groups

By analyzing the distribution tendency of a certain gene in a predetermined gene set, GSEA can be used to evaluate the gene contribution to the phenotype (Zhang et al., 2020a). GSEA and single-sample Gene Set Enrichment Analysis (ssGSEA) analysis were conducted to determine the differences in biological function in the low-risk and high-risk groups. To evaluate the therapeutic efficacy of chemotherapy and targeted agents for HCC in low-risk and high-risk, we used the R packages “ggplot2” and “pRRophetic” to calculate the half inhibitory concentration (IC50) of chemotherapeutic and target therapeutic drugs. This approach uses statistical modeling from the drug sensitivity and gene expression databases, then matches the models to the gene expression data. We obtained the copy number variation (includes deletion and amplification) of seven prognostic RPLs; the copy number variation of the prognostic PRLs was analyzed to investigate the impact of amplification and deletion on overall survival of HCC. We performed differential analysis for amplifications and deletions of prognostic PRLs. In addition, we analyzed potential drugs for prognostic PRLs. LncMAP is a database with relevant lncRNA-TF-gene regulatory network information for more than 20 tumor types, as well as information regarding drug lncRNA (Li et al., 2018). From the LncMAP database, we extracted the lncRNA expression files of drug effects through the reannotation of the CMap database. Drug-lncRNA interactions were obtained using the Spearman’s correlation analysis, and FDR <0.05 was considered significant. Subsequently, we constructed a PRL-drug network.

### Statistical Analysis

All statistical analyses were performed using R (version: 4.0.4) software. Differentially expressed lncRNAs were identified using the Benjamini–Hochberg method. Pearson correlation analysis was performed to select pyroptosis-related lncRNAs, while the OS of the different HCC subtypes was evaluated using Kaplan–Meier curves through the “survivalROC” package. The “heatmap” package was used to depict the correlation of clinicopathological features and the subtypes, and Univariate Cox regression analysis and LASSO analysis were used to construct a prognostic risk model. The 1 -, 3 -, and 5-years predictive accuracy of the prognostic model was assessed using ROC curves. Here, two-sided *p* < 0.05 was considered statistically significant.

## Results

### The Landscape of Genetic Alterations in HCC Samples

**FIGURE 2:**
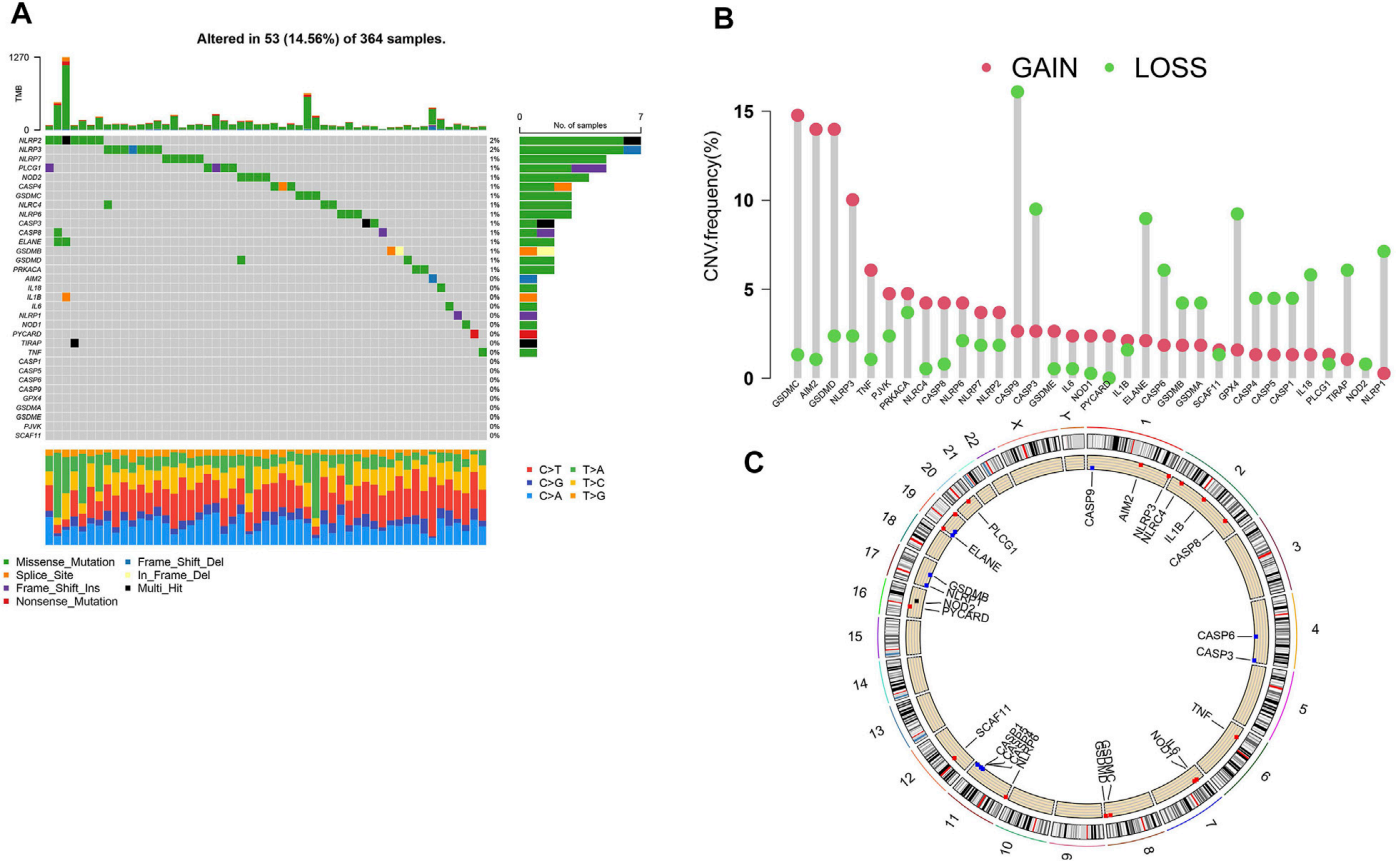
Gene alterations in HCC samples from TCGA. **(A)** The mutations landscape of pyroptosis-related genes in 364 HCC samples of TCGA cohort. The waterfall plot depicts each gene’s mutation information. The annotation represents the mutation type. **(B)** The CNV frequency of pyroptosis-related genes in TCGA database. Red for amplification and blue for deletion. **(C)** The location of pyroptosis-related gene’s CNV modification on chromosomes. CNV: copy number variants.

We obtained the copy number variation profiles of pyroptosis-related genes of HCC in UCSC Xena. As is shown in Figure 2A (14.56%) out of the 364 HCC samples had mutations of pyroptosis-related genes. Copy number amplification had a higher frequency than copy number deletion mutations (Figure 2B), and we depicted changes in pyroptosis-related genes with CNVs on the chromosome (Figure 2C).

### Differential Expression of Pyroptosis-Related Genes and PRLs

We assessed the expression of 33 pyroptosis-related genes between 374 HCC tumors and 50 normal tissues. The visualized heatmaps (Supplementary Figure S1A) indicate clear expression differences in most pyroptosis-related genes between HCC tumor and normal tissues, except for that of *NLRP2*, *GSDMA*, *CASP5*, *CASP1*, *IL18*, *NLRC4*, and *TNF*. Pyroptosis-related genes, such as *PJVK*, *CASP8*, *SCAF11*, and *GPX4*, were significantly upregulated in HCC tumor tissues, whereas *NLRP3* and *IL1B* were upregulated in normal tissues, suggesting that pyroptosis-related genes are crucial in HCC. We constructed a co-expression network between pyroptosis-related genes and lncRNAs (Supplementary Figure S1B). The visualized network plots showed the association of *SCAF11*, *CASP8*, *CASP6*, and *NOD1* with numerous lncRNAs, suggesting that these are hub genes and are pivotal during HCC progression.

**FIGURE 3:**
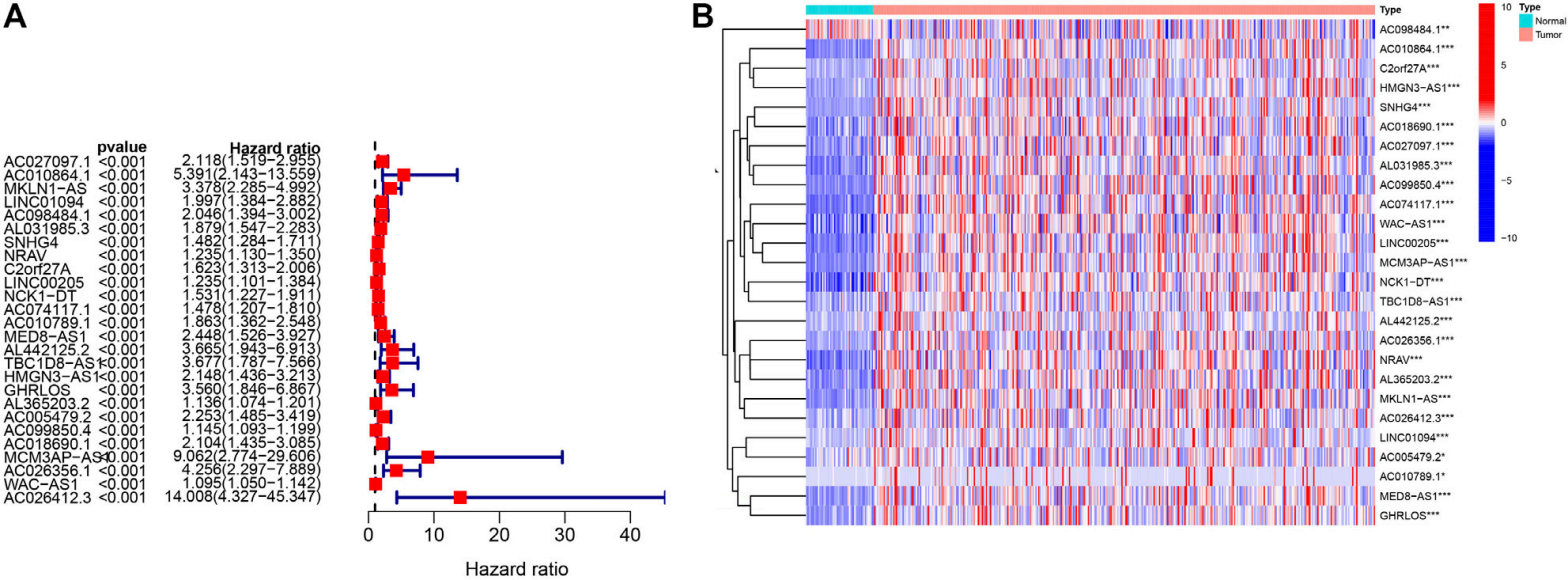
Pyroptosis-related lncRNAs in HCC. **(A)** Univariate Cox regression was performed to screen 26 pyroptosis-related lncRNAs. **(B)** Heatmap presents the 26 pyroptosis-related lncRNAs in HCC tumor tissues and normal tissues. *p < 0.05, **p < 0.01, and ***p < 0.001.

Univariate Cox regression analysis was performed to obtain a signature of 26 PRLs. All 26 PRLs were identified as risk factors (Figure 3A). Except for AC098484.1, the other 25 PRLs were upregulated in HCC tumor tissues compared with that in the normal tissues (Figure 3B).

### Consensus Clustering Analysis

**FIGURE 4:**
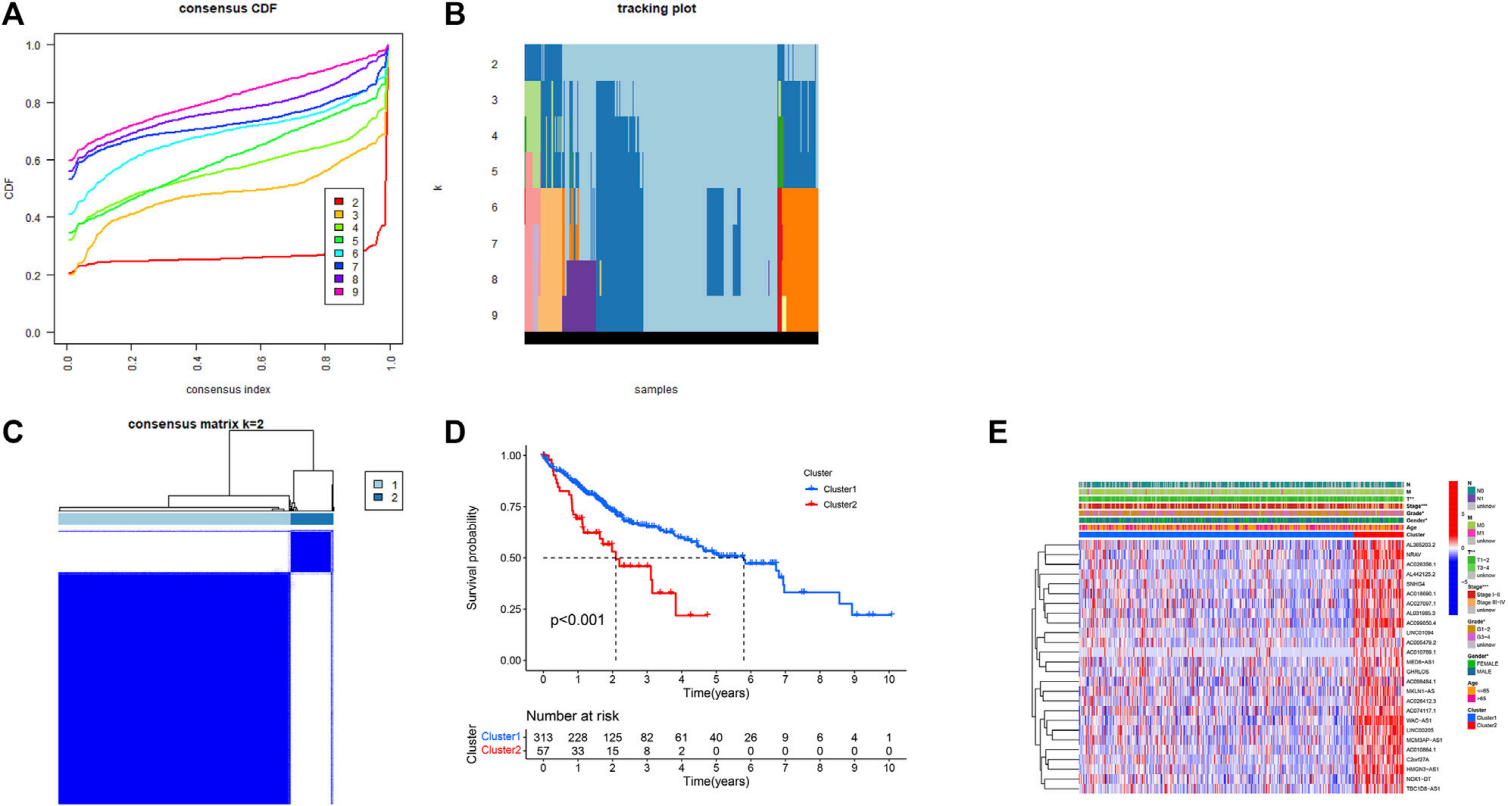
Correlation between the pyroptosis-related lncRNAs and clinicopathological and prognostic features in HCC samples. **(A–C)** Consensus clustering analysis was performed to split the HCC samples into two clusters with k = 2. **(D)** Kaplan–Meier curves of overall survival of HCC patients in the two clusters. **(E)** Heatmap of clinicopathologic features in the two clusters. *p < 0.05, **p < 0.01.

Through Pearson correlation, a total of 770 pyroptosis-related lncRNAs were obtained (Supplementary Table S2), and univariate Cox analysis showed 26 potentially prognostic PRLs concluded in our study (Supplementary Table S3). Consensus clustering analysis was performed with k = 2–9 of the cumulative distribution function. The optimal clustering parameter was identified as k = 2 (Figures 4A–C) based on the expression scale of PRLs and clustering measurements. The HCC cohort was then split into Clusters 1 (*n* = 313) and 2 (*n* = 57) based on the expression of PRLs. Kaplan–Meier curves of OS in Clusters 1 and 2 are shown in Figure 4D. Compared with that of Cluster 2, Cluster 1 had a superior OS (*p* < 0.001). The clinicopathological characteristics of the two clusters were compared and their correlations were explored. The outcome revealed that gender, grade, tumor stage, and T stage were correlated in the two clusters (Figure 4E). Compared with Cluster 1, the prognosis of patients in Cluster 2 was poor, and the tumor pathological grade and stage of HCC patients showed progressive changes. This indicates that the 26 PRLs obtained via univariate analysis may promote tumor progression and metastasis.

### Differences in the Immune Microenvironment Between the Two Clusters and Correlation Analysis

**FIGURE 5:**
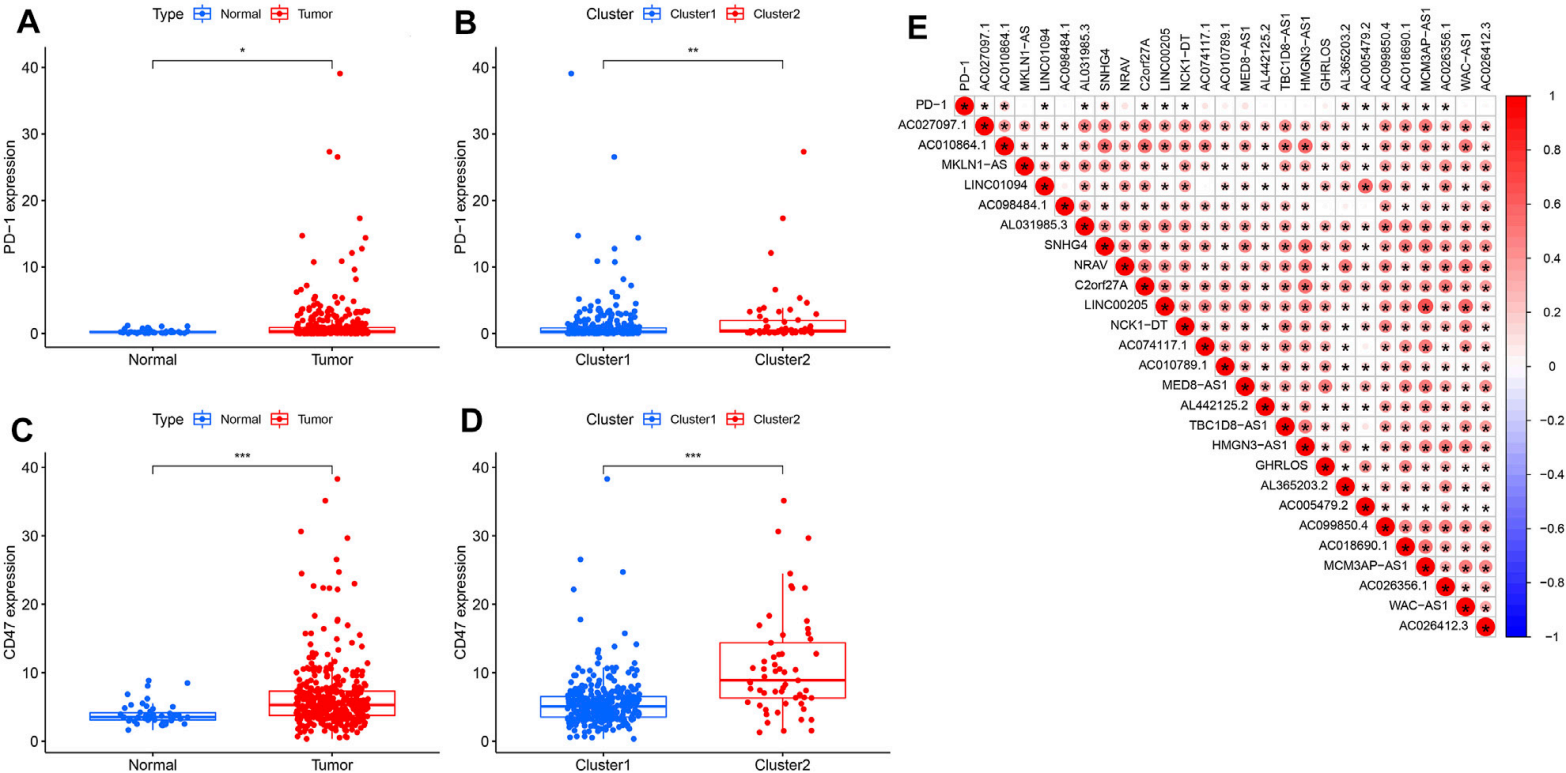
Correlation between PD-L1 and pyroptosis-related lncRNAs. **(A)** Upregulation of PD-1 in the HCC cohort, *p < 0.05. **(B)** PD-1 expression level in cluster 1 and cluster 2 subtypes in the TCGA cohort. **(C)** Upregulation of CD47 in the HCC cohort, ***p < 0.0001. **(D)** CD47 expression in cluster 1 and cluster 2 subtypes. **(E)** Association between pyroptosis-related lncRNAs and PD-L1 expression (the red circle represents a positive correlation).

We detected immune checkpoint-related gene expression in HCC tumors and normal tissues as well as in Clusters 1 and 2. PD-1 expression was upregulated in HCC tumor tissues compared with that in normal tissues (*p* < 0.05) (Figure 5A). Similarly, PD-1 expression levels differed between Clusters 1 and 2 samples, with Cluster 2 showing high PD-1 expression (*p* < 0.01) (Figure 5B). Studies have reported that CD47 expression implies higher tumorigenicity and tumor progression (Cao et al., 2020). Our results revealed that, compared with that in normal tissues, CD47 was upregulated in HCC tumor tissues (*p* < 0.001) (Figure 5C). The expression of CD47 in Cluster 2 exceeded that in Cluster 1 (*p* < 0.001) (Figure 5D). Association analysis revealed that PD-1 expression was positively correlated with AC010864.1, SNHG4, AL365203.2, AC099850.4, and AC018690.1 (Figure 5E).

**FIGURE 6:**
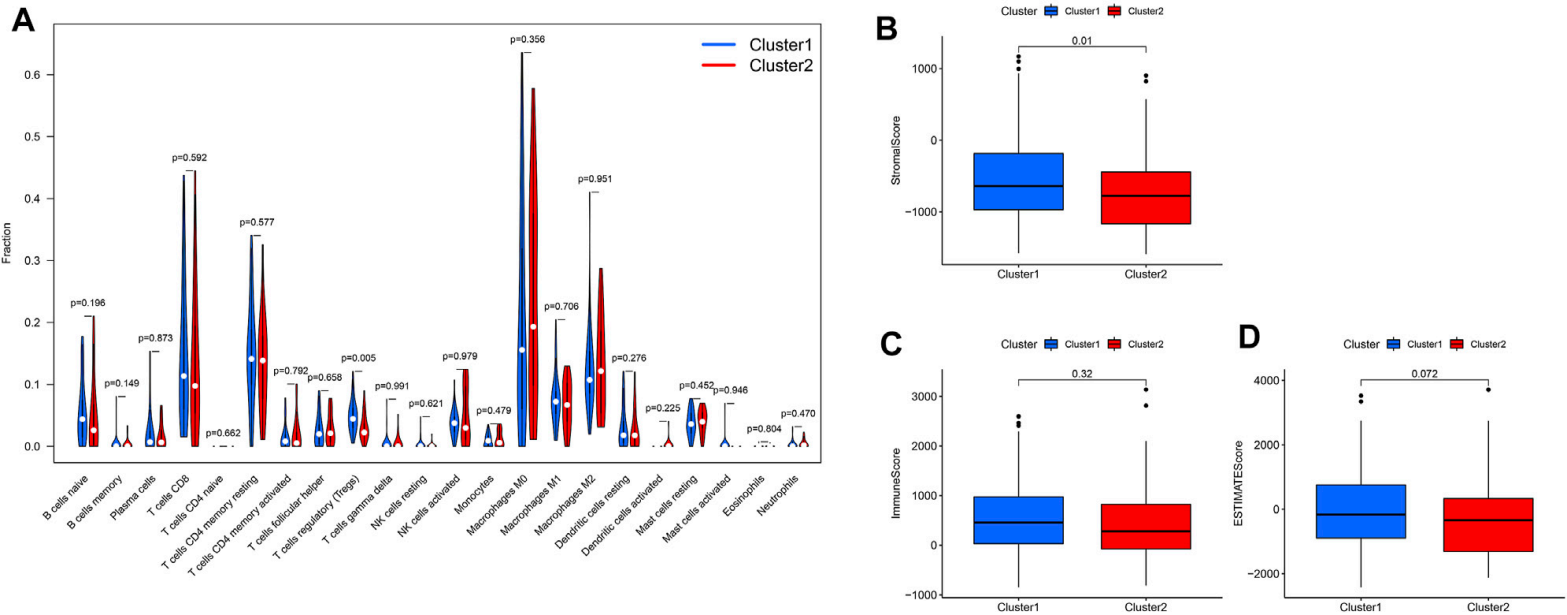
Immune infiltration and stromal scores, immune scores, and estimate scores in cluster 1 and cluster 2 subtypes. **(A)** Infiltration of 22 immune cells in the two clusters. **(B)** Stromal score in clusters 1 and 2. **(C)** Immune score in the two clusters. **(D)** ESTIMATE score in the two clusters.

We calculated the stromal, immune, and estimated scores in the two clusters (Figures 6B–D). The immune score is the overall infiltration score of 22 immune cells in Clusters 1 and 2; although not statistically different in the two cohorts (Figure 6C), it is apparent that the immune score was higher in Cluster 1. In contrast, regulatory T cells were abundant in Cluster 1 (*p* < 0.05) (Figure 6A) when comparing the 22 immune cell infiltrates in the two clusters separately.

### Construction and Validation of the Prognostic Model for HCC

**FIGURE 7:**
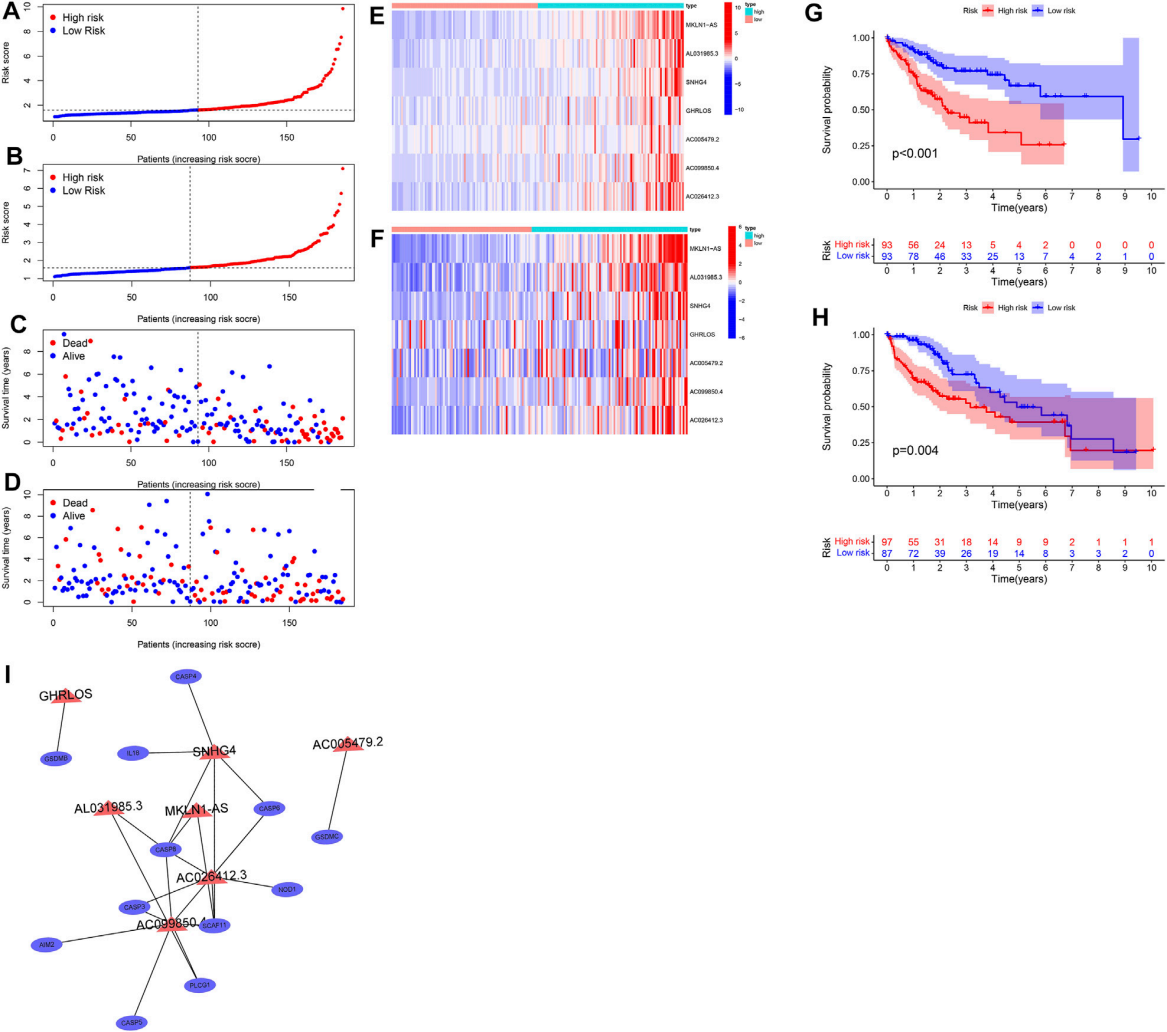
Construction and validation of prognostic signatures of pyroptosis-related lncRNAs. **(A,C)** Distribution of risk score, OS, and OS status in the training cohort. **(B,D)** Distribution of risk score, OS, and OS status in the test cohort. **(E,F)** Heatmap of the signature of seven pyroptosis-related lncRNAs in the training cohort **(E)** and the test **(F)** cohorts. **(G,H)** Kaplan–Meier curves of overall survival for patients with HCC based on the risk score in the training **(G)** and test **(H)** cohorts. Correlation between seven pyroptosis-related lncRNAs and pyroptosis genes **(I)**.

Univariate Cox regression and LASSO analysis were conducted to obtain a seven prognostic PRL signature (Supplementary Table S4) in the training cohort (*n* = 186) based on 26 PRLs. The trajectory of the coefficient of each gene with a value of −ln (lambda) is shown in Supplementary Figure S3. The model was optimal when we chose the seven genes as the signature. Our PRLs model has the following formula: risk score = 0.586267220831113 * MKLN1AS expression +0.186274622429398 * AL031985.3 expression +0.0953167958403102 * SNHG4 expression +0.101802699593077 * GHRLOS expression +0.0732474027666243 * AC005479.2 expression +0.0052148199063163 * AC099850.4 expression +0.703415987615919 * AC026412.3 expression. The risk score and survival status in both the training and test sets (Supplementary Table S5) are presented in Figures 7A–D. As the risk score increased, so did the proportion of patients in the high-risk group, along with the mortality levels rose. The visualized heatmap revealed that the seven PRLs were highly expressed in high-risk groups in the training and test cohorts (Figures 7E,F). As represented by the Kaplan–Meier curves, the OS of the low-risk group tended to be greater than that of the high-risk group in both the training (*p* < 0.001) (Figure 7G) and test sets (*p* = 0.004) (Figure 7H). The 1-, 3-, and 5-years AUC values of the ROC curve were 0.748, 0.717, and 0.714 in the training set and 0.763, 0.621, and 0.616 in the test set, respectively (Supplementary Figures S2A–F). Similarly, we unveiled the association between the seven PRLs and pyroptosis genes, as shown in Figure 7I. LncRNA SNHG4 expression was closely related to that of *IL-18*, *CASP4*, *CASP6*, and *CASP8*, which are the hub genes.

**FIGURE 8:**
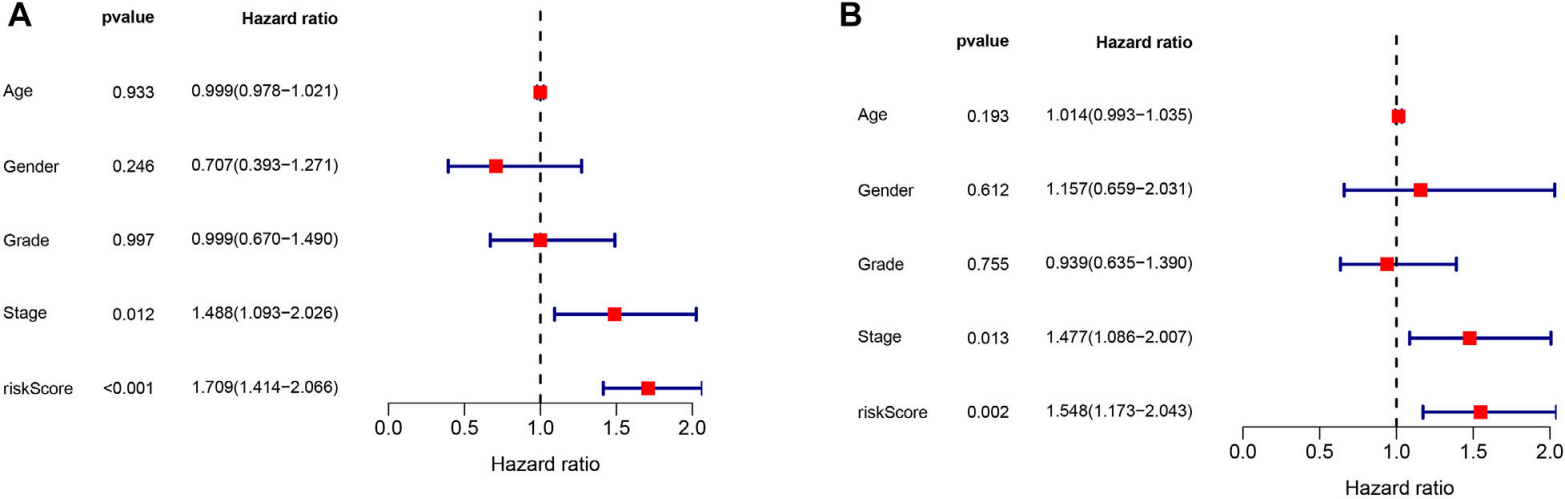
Multivariate Cox regression analysis in both the training and test cohorts. **(A)** Multivariate Cox regression analysis in the training cohort. **(B)** Multivariate Cox regression analysis in the test cohort.

As shown in Figure 8A, multivariate Cox regression of the HCC cohort revealed that both tumor stage (*p* = 0.012, HR = 1.488, 95% CI: 1.093–2.026) and risk score (*p* < 0.001, HR = 1.709, 95% CI: 1.414–2.066) were independent risk factors for HCC prognosis in the training set. Multivariate Cox regression on the test set (Figure 8B) also indicated that tumor stage (*p* = 0.013, HR = 1.477, 95% CI: 1.086–2.007) and risk score (*p* = 0.002, HR = 1.548, 95% CI: 1.173–2.043) were independent risk factors for HCC prognosis.

### Correlation Between Risk Scores and Clinicopathological Factors in HCC

**FIGURE 9:**
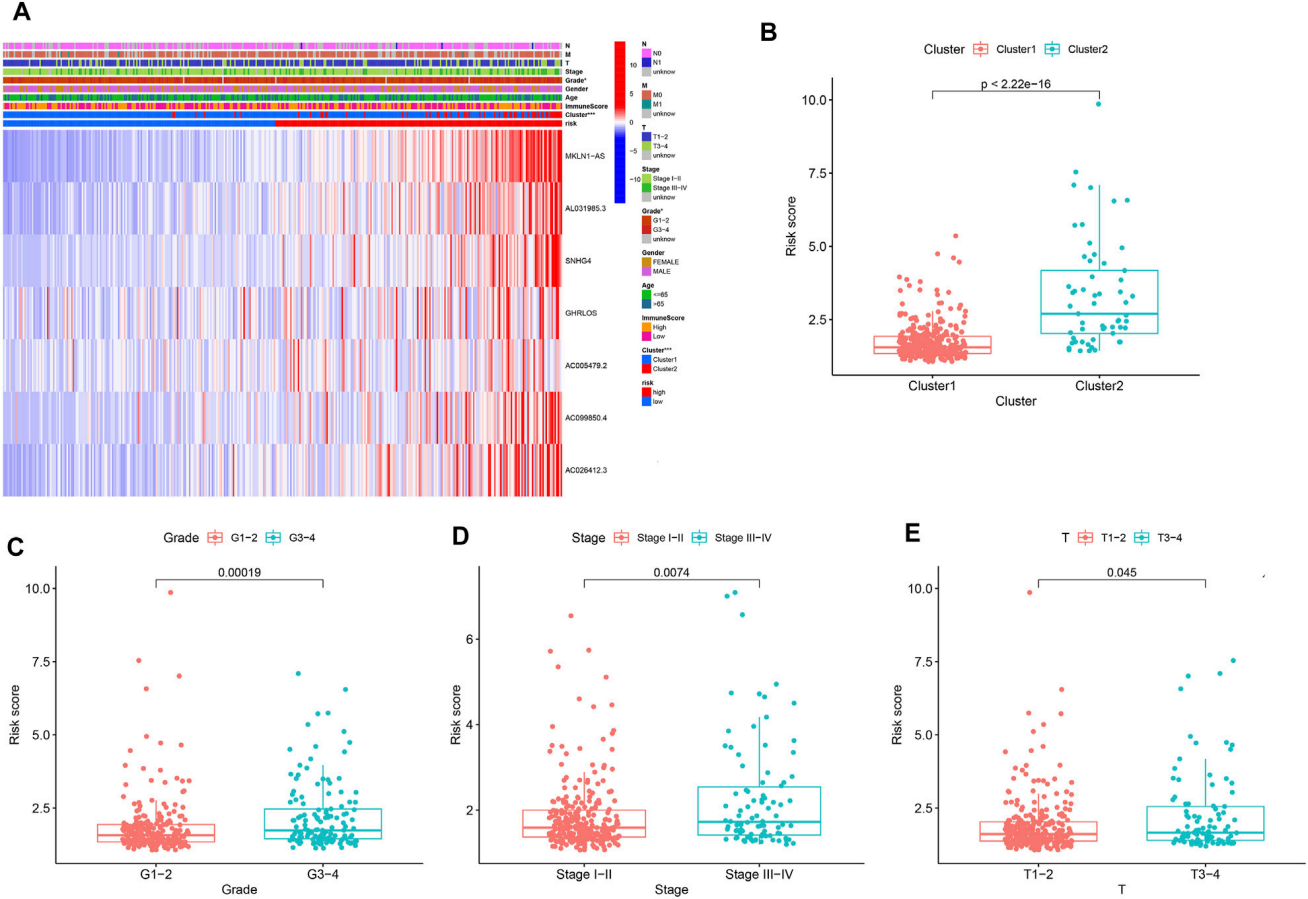
Prognostic risk scores correlated with clinicopathological features in TCGA cohorts. **(A)** Heatmap and clinicopathologic features of high-risk and low-risk groups. *p < 0.05, **p < 0.01, and ***p < 0.001. **(B–E)** Distribution of risk scores stratified by cluster **(B)**, grade **(C)**, stage **(D)**, and T stage **(E)**.

The heatmap (Figure 9A) depicts the correlation between clinicopathological characteristics and prognosis risk signature. The seven PRLs were highly expressed in the high-risk group compared with the low-risk group, suggesting that the seven PRLs were risk factors. In addition, the risk scores in Cluster 2 (*p* < 2.22e-16, Figure 9B), G3–4 (*p* = 0.00019, Figure 9C), Stage III-IV (*p* = 0.0074, Figure 9D), and T III–IV (*p* = 0.045, Figure 9E) were high. This illustrates the superior ability of a risk score composed of seven PRLs to discriminate among subgroups of clinicopathological features.

**FIGURE 10:**
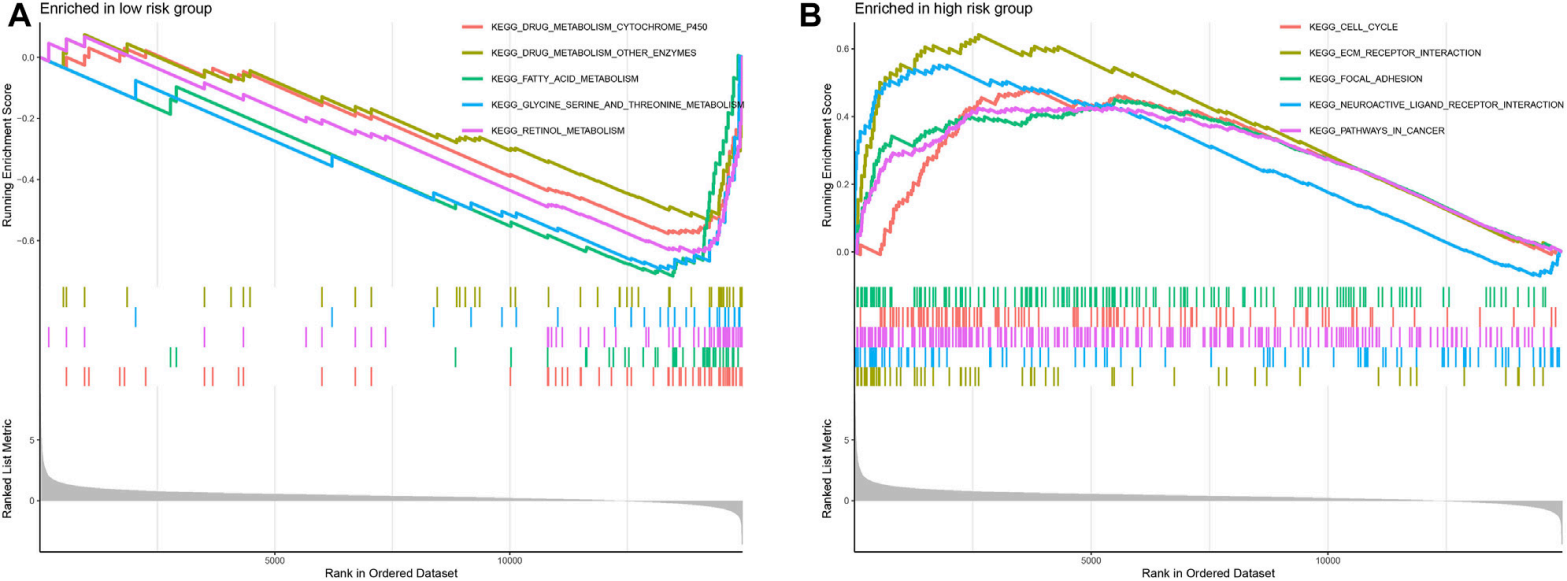
Gene set enrichment analysis (GSEA) in low-risk and high-risk groups. **(A)** GSEA of the top five signature pathways in the low-risk group. **(B)** GSEA of the top five signature pathways in the high-risk group.

GSEA showed that the top five signature pathways ranked were “metabolism cytochrome,” “metabolism other enzymes,” “fatty acid metabolism,” “glycine serine and threonine metabolism,” and “retinol metabolism” in the low-risk group (Figure 10A) and “cell cycle,” “ecm receptor interaction,” “focal adhesion,” “neuroactive ligand-receptor interaction,” and “pathways in cancer” in the high-risk group (Figure 10B).

### Immune Status and Immune Function in the Low-Risk and High-Risk Groups

**FIGURE 11:**
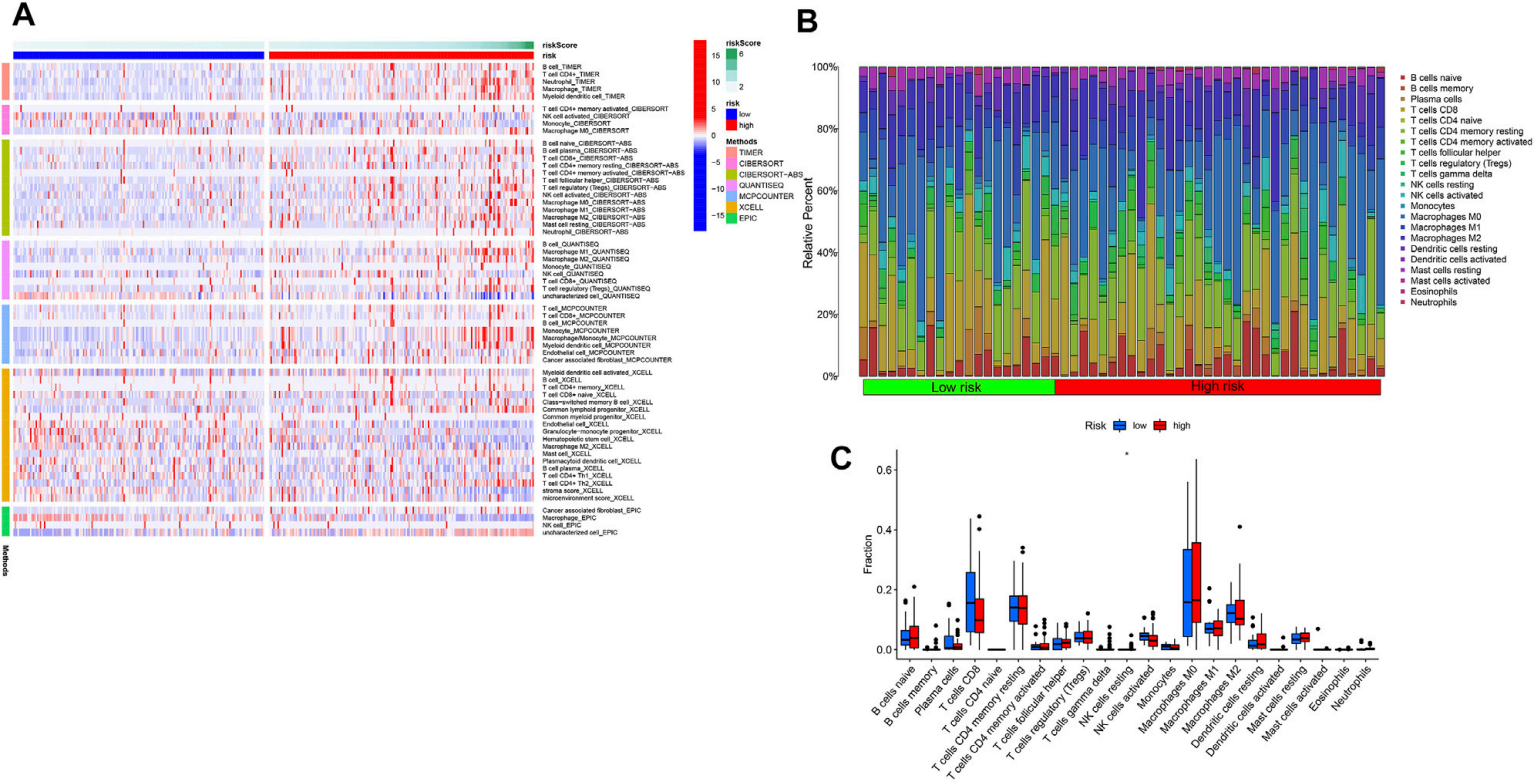
Immune cell infiltration landscape in HCC. **(A)** Heatmap for immune cell infiltration landscape based on the CIBERSORT, CIBERSORT, ABS, QUANTISEQ, XCELL, MCPcounter, EPIC, and TIMER algorithms among high-risk and low-risk groups. **(B)** Bar plot of the immune infiltration cell proportions based on the CIBERSORT algorithm in the low-risk and high-risk groups. **(C)** Bar plot of the distribution of the infiltration of 22 immune cells in the low-risk and high-risk groups.

**FIGURE 12:**
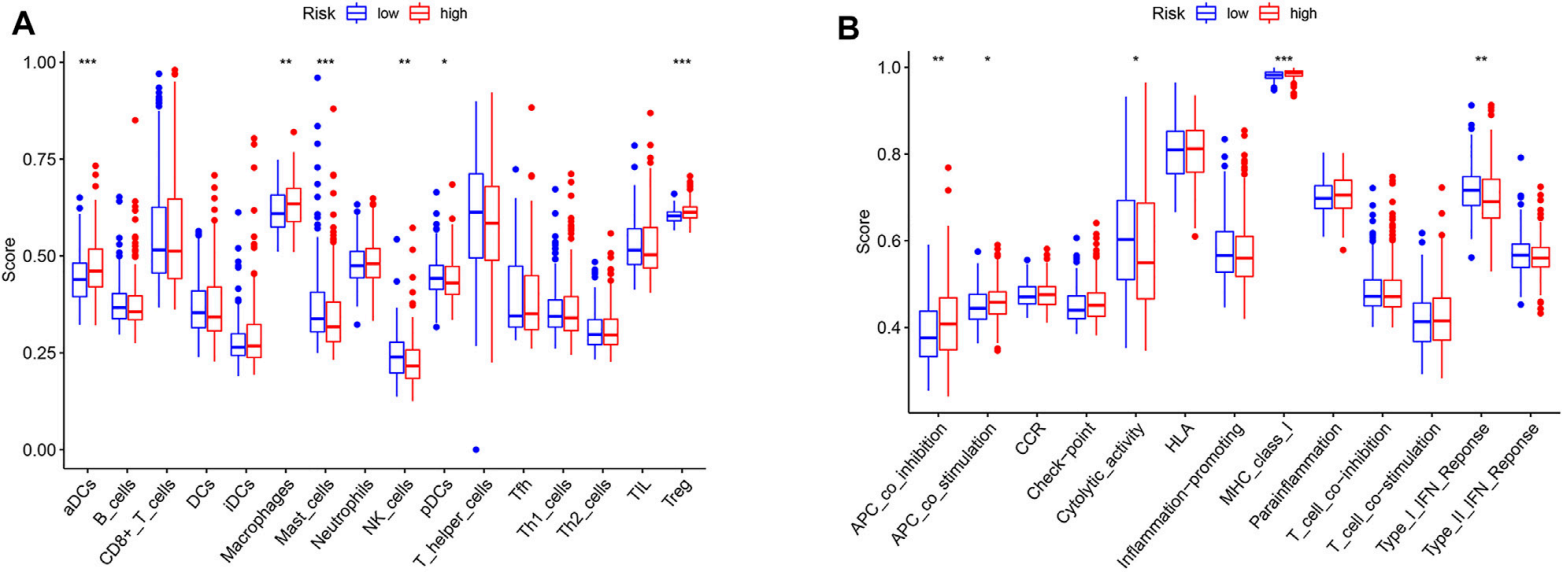
Bar plot showing that the low-risk and high-risk groups exhibit different immune and pyroptosis statuses. **(A)** Single sample gene set enrichment analysis (ssGSEA) for the immune functions between high and low HCC risk groups. **(B)** Expression levels of immune function signature between high and low HCC risk groups. (*p < 0.05, **p < 0.01, and ***p < 0.001).

Based on the risk score of seven PRLs, the proportion of tumor-infiltrating immune cells in the low-risk and high-risk groups are shown in Figures 11A,B. Both CD4^+^ T and CD8^+^ T cells were highly infiltrated in the high-risk group compared to the low-risk group. The differences in the expression of pyroptosis genes are shown in Figure 11C. *GPX4* (*p* < 0.01) and *NLRP6* (*p* < 0.001) were highly expressed in the low-risk group, whereas the others were upregulated in the high-risk group. ssGSEA results revealed that immune-related responses were largely enriched, and immune cells, including activated dendritic cells (aDCs), macrophage cells, and regulatory cells (Tregs), were highly activated in the high-risk group (*p* < 0.01) (Figure 12A). The cytolytic activity and type I IFN response were highly enriched in the low-risk group (*p* < 0.05) (Figure 12B).

### Drug Sensitivity Analysis and Exploration of Potential Therapeutic Agents for HCC

**FIGURE 13:**
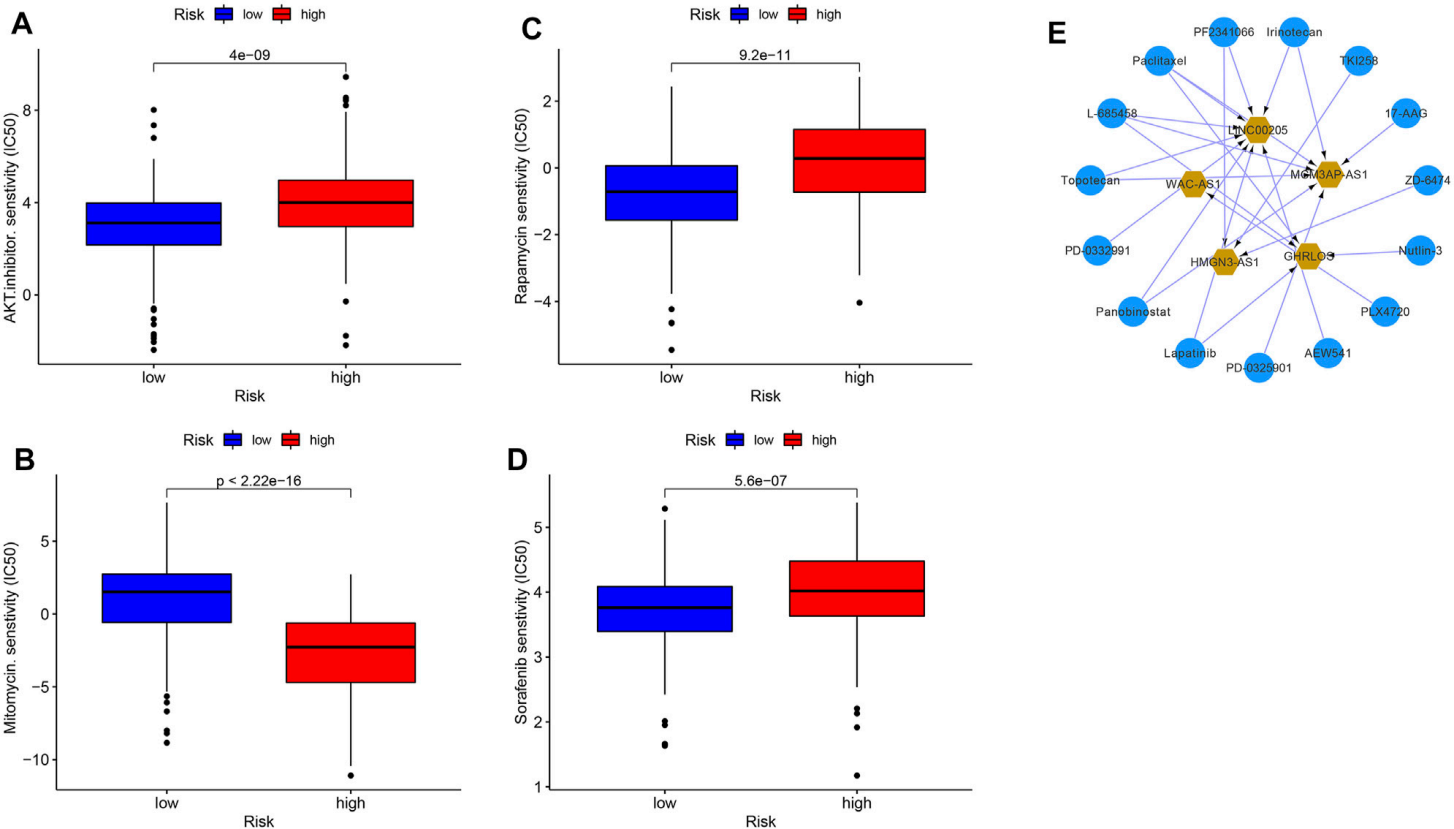
Drug sensitivity in different risk groups and potential targeted drugs. AKT inhibitors **(A)**, mitomycin **(B)**, rapamycin **(C)**, sorafenib **(D)**, and targeted drugs **(E)**.

We analyzed the half maximal inhibitory concentration (IC50) of AKT inhibitors, mitomycin, rapamycin, and sorafenib between the high- and low-risk groups. As shown in Figures 13A–D, except for the IC50 value of mitomycin, which was lower in the high-risk group (*p* < 2.22e-16), the drugs AKT inhibitors (P = 4e-9), rapamycin (*p* = 9.2e-11) and sorafenib (*p* = 5.6e-7) were all sensitive to the low-risk group. It also confirmed that grouping based on PRLs could screen a suitable population for chemotherapy and targeted therapy. A total of 304,303 drug-lncRNA interaction pairs were obtained from the drug-lncRNA module of the lncMAP database (Supplementary Table S6). Profiling 26 prognosis-related lncRNAs obtained a total of 15 lncRNA-related therapeutic agents. This network was composed of five lncRNAs and 15 small-molecule drugs. The top five PRL drugs were Lapatinib, Panobinostat, PD-0332991, Panobinostat, and topotecan (Figure 13E). This may indicate that small-molecule drugs can affect HCC tumor progression and prognosis by acting on the GHRLOS.

### Copy Number Variation Analysis of PRLs

**FIGURE 14:**
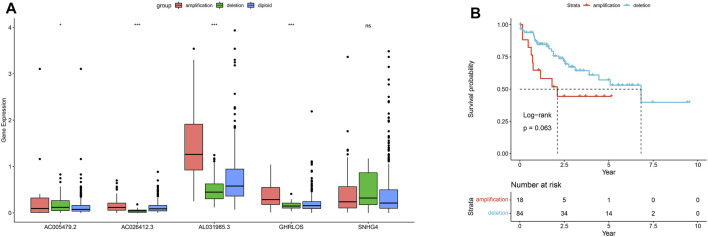
Copy number variations of pyroptosis-related lncRNAs. Copy number amplification, deletion, and ploidy of five pyroptosis-related lncRNAs **(A)**. Kaplan–Meier curves for copy number amplification and deletion survival of AL031985.3 **(B)**.

We obtained CNV data for five of the seven PRLs: AC005479.2, AC026412.3, AL031985.3, GHRLOS, and SNHG4. In addition to SNHG4, the amplification, deletion, and diploid proportion of the remaining four PRLs were significantly different (Figure 14A). The copy number amplification level of AL031985.3 significantly increased (*p* < 0.001). Survival analysis of AL031985.3 CNV showed that the prognosis of samples exhibiting its amplification was worse than that of those with copy number deletion (Figure 14B). This suggests that the copy number amplification of AL031985.3 promotes tumor progression and metastasis.

## Discussion

HCC, a notorious malignancy with complex and heterogeneous tumor microenvironment, is associated with increased morbidity and mortality (Forner et al., 2018; Feng et al., 2021). Although several breakthroughs have been made in diagnosing and treating HCC in recent years, its prognosis remains poor (Yang et al., 2019). Therefore, new prognostic models and individualized decision-making plans are required for HCC treatment. Pyroptosis is a proinflammatory programmed cell death mediated by cysteine–aspartate-specific protein kinases 1, 4, 5, and 11. The process relies on the Gasdermin family of proteins and is characterized by cell swelling, plasma membrane perforation, and the release of small inflammatory molecules, which ultimately induces inflammation (Galluzzi et al., 2018). Pyroptosis is involved in the initiation and progression of several diseases, including cancers, and, in particular, has a dual effect of promoting and inhibiting the formation of the tumor microenvironment (Rathinam and Fitzgerald, 2016). Recently, the regulatory role of lncRNAs in gene transcription and expression has gained increasing attention.

In this study, we identified 770 PRLs using Pearson’s correlation analysis. Subsequent univariate Cox regression analysis was performed to filter out the signature encompassing 26 PRLs, which were considered as potential prognosis-related signatures. Based on this, we used a consensus clustering algorithm to classify HCC samples into Clusters 1 and 2. Through LASSO regression analysis, we screened seven prognosis-related signatures from 26 PRLs. The HCC cohorts in the training and test sets were divided into high-risk and low-risk groups based on the median values of the seven PRLs. The AUC of ROC shows the superior predictive efficacy of this prognostic signature for the OS of patients with HCC. In addition, we analyzed the immune checkpoint PD-1 and CD47 and demonstrated their high expression in tumor tissues compared with normal tissues, which shows the feasibility of anti-PD-1 and anti-CD47 antibodies in the immunotherapy of HCC. Moreover, further immune checkpoint blockage targeting PD-1 and CD47 can benefit more HCC patients. In previous studies, Zhang et al. have found that cancer associated fibroblasts (CAFs) in the tumor microenvironment promote extracellular matrix (ECM) remodeling and angiogenesis, while enhancing immunosuppression in liver cancer, providing a theoretical basis for developing targeted drugs against CAFs (Zhang et al., 2020b). In addition, the authors demonstrated that cyclin dependent kinase regulatory subunit 2 (CKS2) in the cell cycle dependent protein kinase subunit family, as a novel tumor biomarker, is highly expressed in HCC and that its elevated expression is predictive of poorer prognosis and tumor progression (Zhang et al., 2019). Conversely, we classified HCC patients into either high- or low-risk groups based on prognostic PRLs signature and showed that PD-1 and CD47 were highly expressed in tumor tissues, which may serve as candidate immune checkpoints. This provides a reference for clinical screening of suitable patients for immune and targeted therapies.

*CASP8*, *CASP6*, *GSDME*, *NOD1*, and GPX4 were significantly upregulated in tumor tissues compared to the normal tissues, whereas *IL6*, *IL1B*, *NLRP3*, and *NLRC4* were downregulated, suggesting that pyroptosis-related genes play important roles in tumor progression and prognosis. Univariate Cox regression analysis of the PRLs yielded 26 negatively correlated signatures with prognosis. Consensus clustering analysis was performed to obtain two clusters. The role of N6-methyladenosine (m6A)-associated lncRNAs in immune infiltration and prognosis of HCC have been previously illustrated by Wang et al. (Wang et al., 2021a), who similarly divided the TCGA liver cancer cohort into Clusters 1 (*n* = 313) and 2 (*n* = 57) based on consensus clustering analysis; Cluster 1 had a superior prognosis compared to Cluster 2 (*p* < 0.001). This is consistent with our analysis, where Cluster 1 had a better prognosis than Cluster 2 in our cohort (*p* < 0.001) (Figure 4B). In contrast to the study of Wang et al., we analyzed *PD-1* expression in both the tumor and normal groups, Clusters 1 and 2; the results show that PD-1 was upregulated in the tumor group, providing the theoretical basis and prospect of applying anti-PD-1 antibody in the treatment of patients with HCC. In addition, the 22 immune cell infiltrations in the two clusters were compared, and the results demonstrate that the degree of T cell regulatory infiltration differed between the two groups, with more of Cluster 1 (*p* < 0.005) than Cluster 2. Therefore, it can be speculated that the genes in Cluster 1 responded effectively to immune checkpoint inhibitors.

In the present work, a signature based on the expression of seven PRLs was obtained by LASSO regression analysis. Several lncRNAs of this signature have been previously studied. For instance, Wang et al. (2021b) constructed an HCC prognostic risk model including AL031985.3 as a prognosis-related risk factor, and its expression in the high-risk group exceeded that in the low-risk group. In our study, AL031985.3 was similarly a risk factor for HCC prognosis, and its expression was significantly upregulated in the high-risk group. Zhou et al. (2021) constructed a liver cancer prognostic signature composed of three hypoxia-related lncRNAs, and the correlation between the three hypoxia-related lncRNAs and genes was similarly explored. In our study, the correlation of seven PRLs with pyroptosis genes was similarly analyzed; the visualized co-expression network is presented in Figure 7I.

Although the association of PRLs with breast cancer and clear cell carcinoma has been investigated (Gao and Li, 2021; Tang et al., 2021), the role of these lncRNAs in HCC remains to be elucidated. Our study revealed the association of seven PRLs with HCC immune infiltration, immune checkpoint gene expression, and prognosis, and this signature was able to discriminate between different tumor stages and pathological grades.

Recently, immunotherapy has attracted increasing attention, and anti-PD-1 antibodies have shown definite efficacy against various solid tumors, such as breast and small cell lung cancers (Voorwerk et al., 2019; Kroemer and Kepp, 2021). Several studies have reported that, for unresectable HCC, PD-1/PD-L1 inhibitors with antiangiogenic-targeted agents achieve significant objective responses and disease control rates (Finn et al., 2020a; Finn et al., 2020b; Xu et al., 2021). In our study, the immune checkpoint gene PD-1 was upregulated in the HCC tumor group compared with that in normal tissue and is thus suitable for immunotherapy with an anti-PD-1 blockade. ssGSEA analysis showed the differences between immune cells and immune function in the high- and low-risk groups. The low-risk group had greater infiltration of pDCs, mast cells, and NK cells, whereas the high-risk group had more macrophages, Tregs, and aDCs.

Su et al. used multi-omics analysis to identify biomarkers of brain metastatic tumors and derived prognostic subtypes (Su et al., 2020), while Song et al. constructed prognostic subnetwork signatures (SPNs) for metastatic breast cancer (Song et al., 2015). In the present study, we delineated the mutation spectrum of 33 pyroptosis related genes in TCGA, and copy number variation frequencies. The amplification frequency of pyroptosis-related genes was higher than the deletion frequency. CNV analysis of seven prognostic PRLs signatures showed that AL031985.3 had a worse prognosis with copy number amplification than with copy number deletion. Therefore, altered prognostic PRLs copy number amplification may potentially improve HCC prognosis.

In this study, we constructed a prognostic PRL signature with superior predictive precision for patients with HCC. The AUC of ROC was 0.748, 0.717, and 0.714 in the training set and 0.763, 0.621, and 0.616 in the test set at 1, 3, and 5 years, respectively. Based on the median value of the seven PRLs, HCC patients were categorized into either high- or low-risk groups. The OS of the low-risk group exceeded that of the high-risk group in both the training and test sets. In addition, we analyzed seven PRLs and protein-coding genes and constructed a co-expression network of PRLs and protein-coding genes. Correlation analysis was performed between PRLs and the HCC tumor microenvironment, immune cell infiltration, and immune checkpoint genes to obtain two HCC subtypes; the immune infiltration of Cluster 1 was better than that of Cluster 2. This indicates that the seven PRLs play an indispensable role in the subtype analysis and prognosis of HCC. Our study provides insights into immunotherapy targeting PD-1 and CD47.

## Conclusion

In our study, we systematically evaluated the prognostic value of PRLs, their association with PD-1 effects on the tumor microenvironment. A deeper understanding of the association of PRLs in the tumor microenvironment can potentially improve the efficacy of immunotherapy for HCC.

## Data Availability

The original contributions presented in the study are included in the article/Supplementary Material, further inquiries can be directed to the corresponding author.
