# Supplementary material for: Comprehensive Analysis of the Correlation Between Pyroptosis-Related LncRNAs and Tumor Microenvironment, Prognosis, and Immune Infiltration in Hepatocellular Carcinoma
Source: Front Genet. 2022 Apr 26;13:867627. doi: 10.3389/fgene.2022.867627 (PMC9087742; doi:10.3389/fgene.2022.867627)
Supplement: Supplementary file 1 [file DataSheet1.docx]

Supplementary Tables

Supplementary Figure 1 (A-B) Expression characteristics and correlation of pyroptosis-related genes in hepatocellular carcinoma (HCC). (A) Heatmap of the expression of 33 pyroptosis-related genes in HCC tumor tissue (T) and normal tissues (N) from The Cancer Genome Atlas (TCGA) datasets. *P < 0.05, **P < 0.01, and ***P < 0.001. (B) Interaction network of pyroptosis-related genes (blue) and associated lncRNAs (yellow).


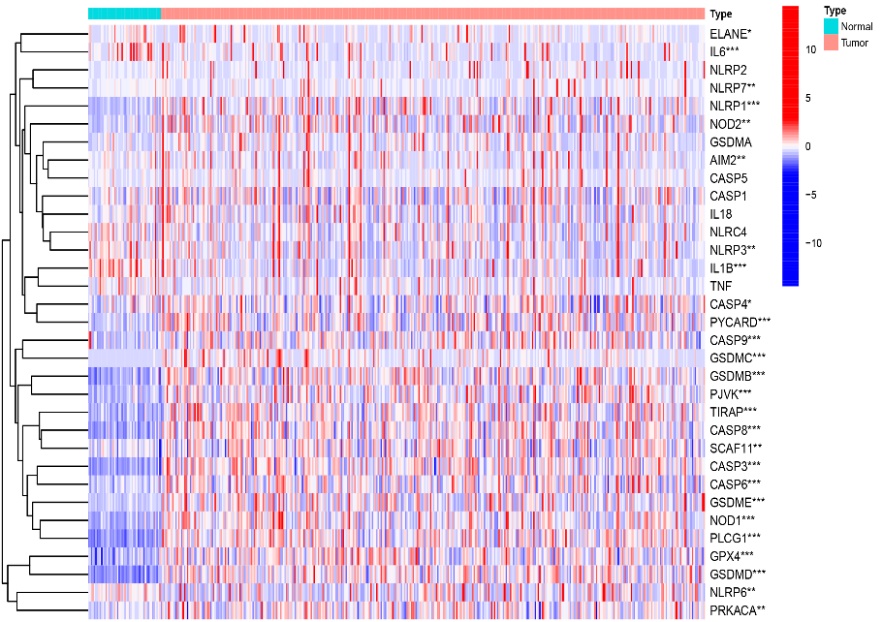


A


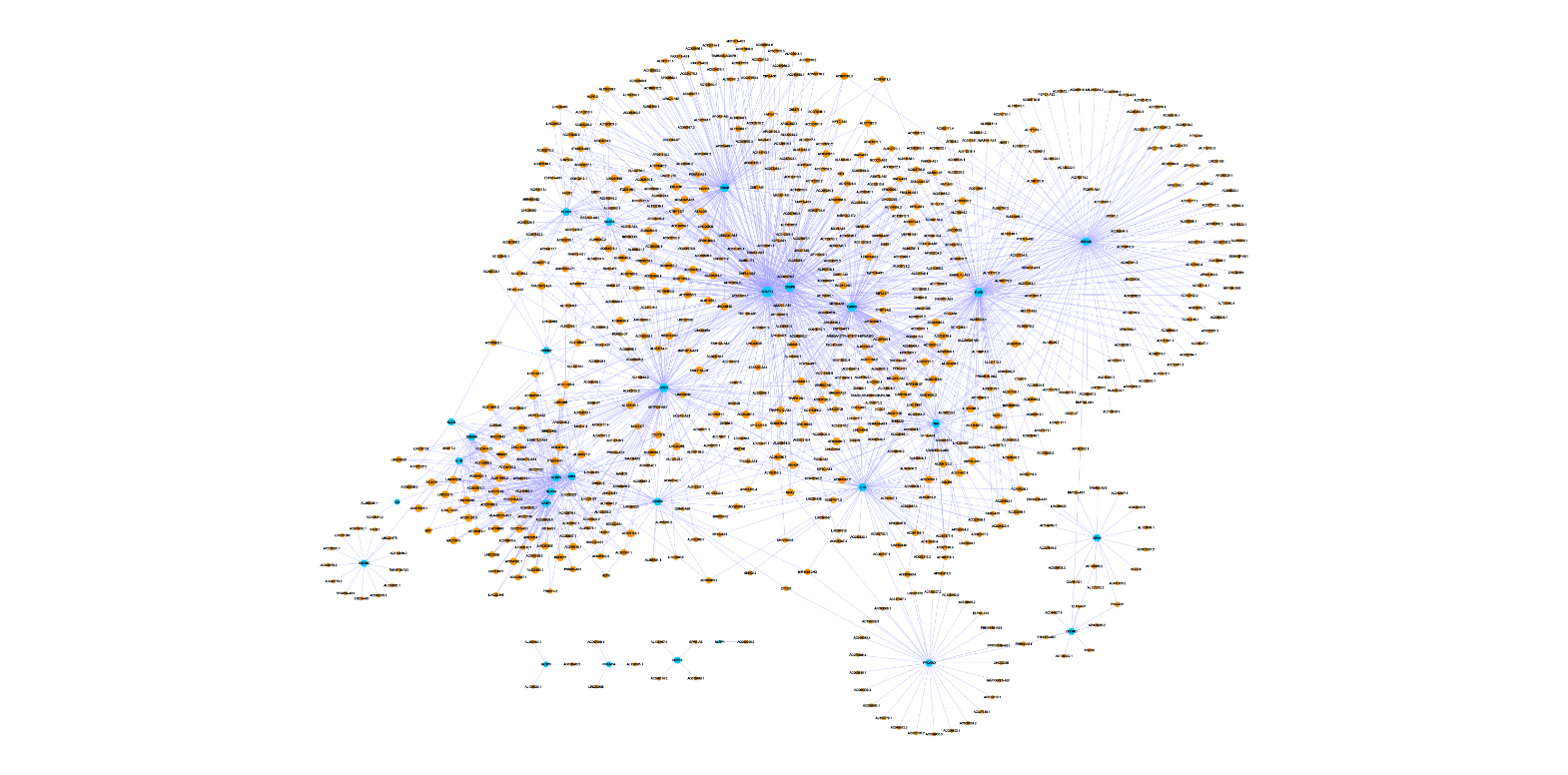


B

Supplementary Figure 2 (A-F) The 1, 3, and 5 year area under the curve (AUC) of receiver operating characteristic (ROC) in train set (A, B and C) and test set (D, E and F) of HCC patients


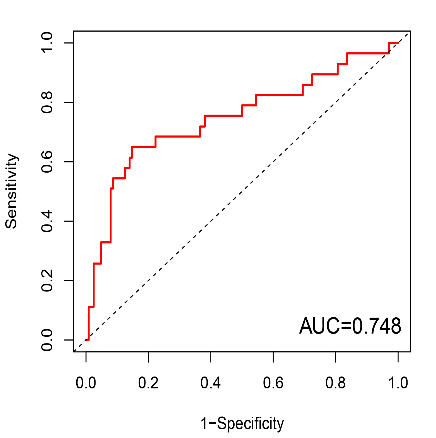

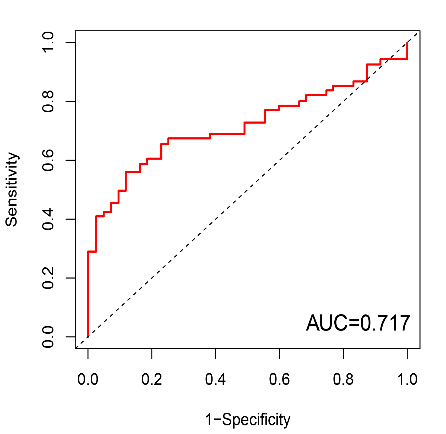

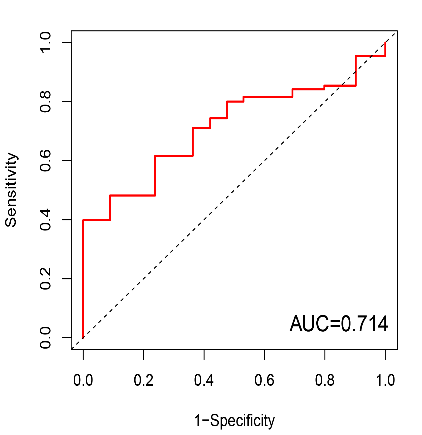


A B C


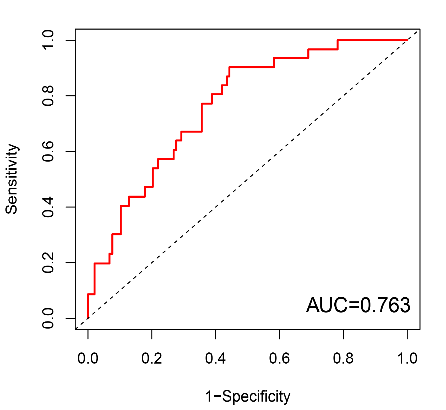

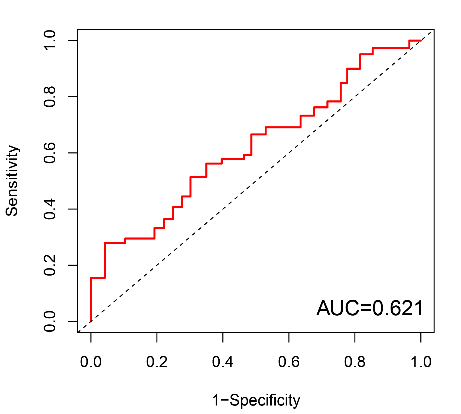

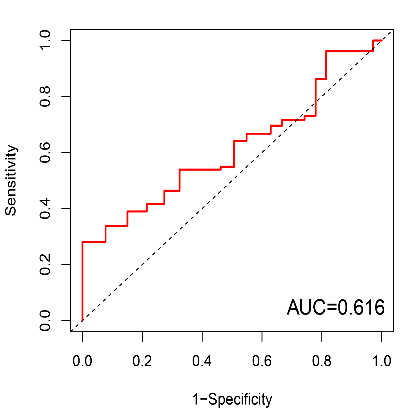


D E F

Supplementary Figure 3 LASSO based on pyroptosis-related genes and prediction effect in the training set. (A) The trajectory of each variable; (B) The confidence interval of each lambda.


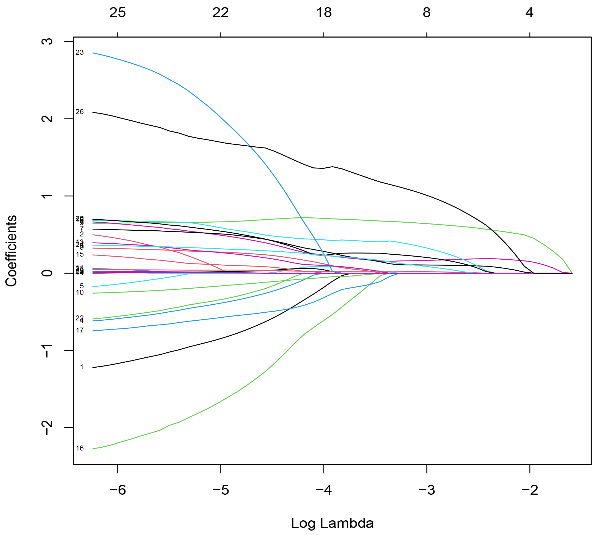

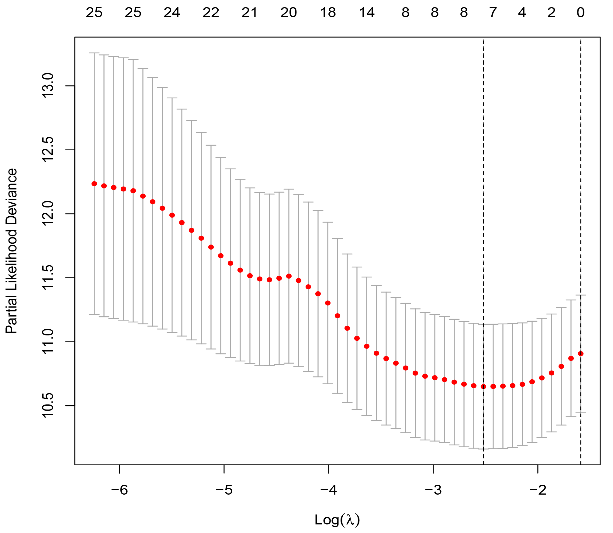


A B
